# Supplementary material for: Heparan sulfate is essential for Drosophila FGF export
Source: bioRxiv. 2026 Mar 26:2026.03.24.714045. Preprint. [Version 1] doi: 10.64898/2026.03.24.714045 (PMC13041799; doi:10.64898/2026.03.24.714045)
Supplement: Supplementary file 1 [file NIHPP2026.03.24.714045v1-supplement-1.pdf]

bioRxiv preprint doi: https://doi.org/10.64898/2026.03.24.714045; this version posted March 26, 2026. The copyright holder for this preprint (which was not certified by peer review) is the author/funder, who has granted bioRxiv a license to display the preprint in perpetuity. It is made available under aCC-BY 4.0 International license.

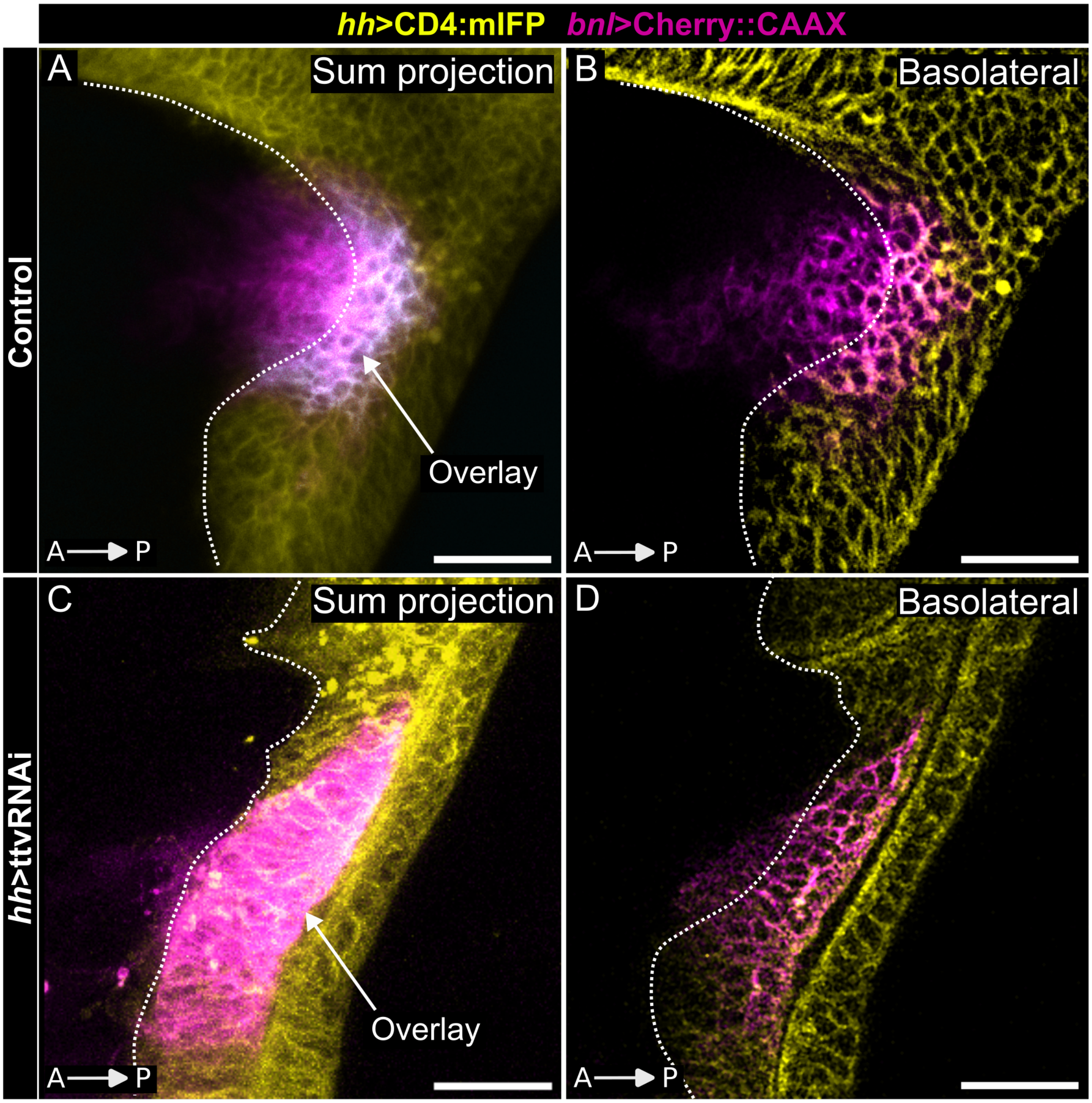

Supplementary Figure 1: Bnl-producing domain straddles A/P compartment border

(A-), Wing disc with Bnl-producing cells expressing Cherry:CAAX (*bnl*-Gal4>UAS-Cherry:CAAX – magenta) and P compartment cells expressing mIFP (*hh*-Gal4>UAS-CD4:mIFP). (A,C) Sum projection of confocal stacks, and (B,D) basal lateral views showing Bnl-producing cells (magenta) and P compartment (yellow); overlay (white); A/P compartment border (dashed line). (A,B) controls; (C,D) disc also expressing ttvRNAi in dorsal compartment (*ap*-Gal4>UAS-ttvRNAi).

bioRxiv preprint doi: https://doi.org/10.64898/2026.03.24.714045; this version posted March 26, 2026. The copyright holder for this preprint (which was not certified by peer review) is the author/funder, who has granted bioRxiv a license to display the preprint in perpetuity. It is made available under aCC-BY 4.0 International license.

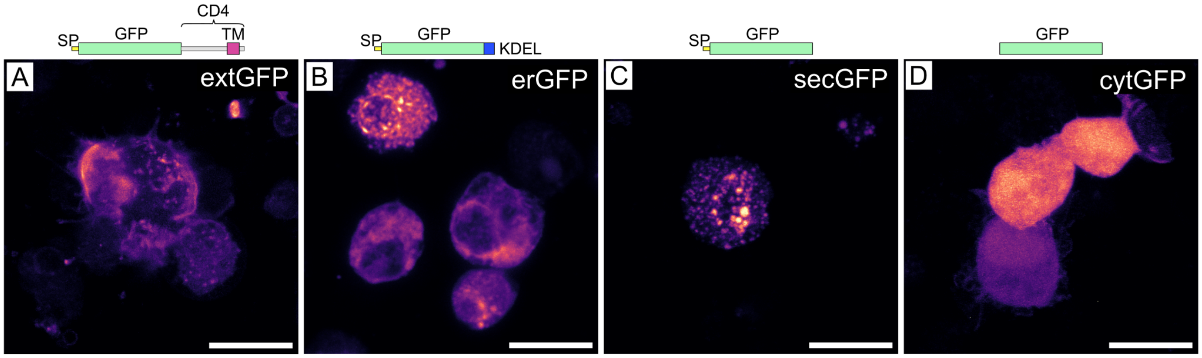

Supplementary Figure 2: Subcellular targeting GFP in S2 cells

S2 cells expressing: (A) extGFP (GFP linked to a N-terminal signal peptide (SP) and C-terminal CD4 transmembrane domain (TM)) localizes to plasma membrane; (B) erGFP (erGFP linked to N-terminal SP and a C-terminal KDEL sequence) localizes to ER; (C) secGFP (GFP with N-terminal SP) localizes in cytoplasmic puncta; (D) cytGFP (GFP alone) localizes in cytoplasm.

bioRxiv preprint doi: https://doi.org/10.64898/2026.03.24.714045; this version posted March 26, 2026. The copyright holder for this preprint (which was not certified by peer review) is the author/funder, who has granted bioRxiv a license to display the preprint in perpetuity. It is made available under aCC-BY 4.0 International license.

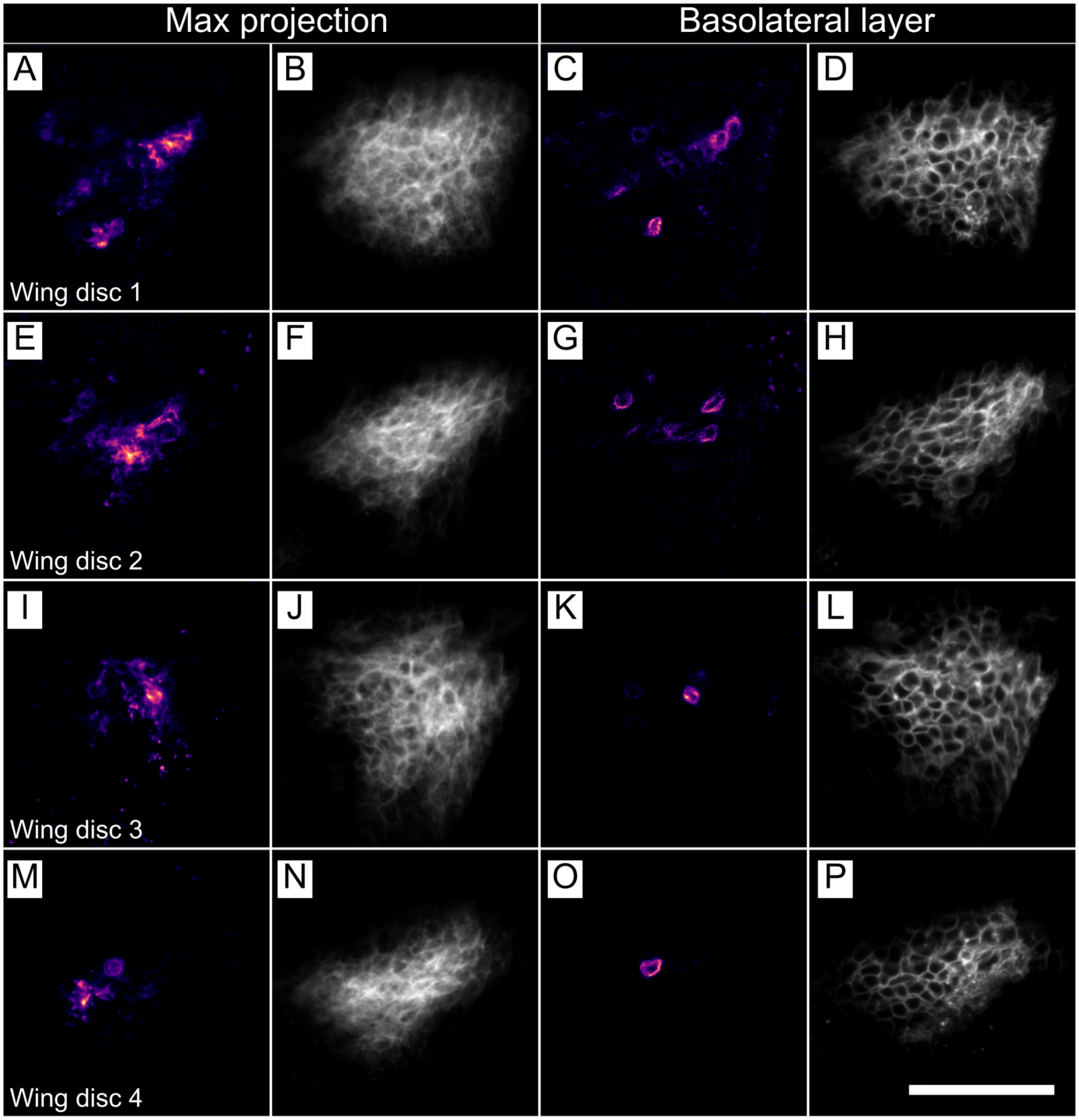

Supplementary Figure 3: Cells with external Bnl in the Bnl-expressing domain of the wing disc

Four wing discs, genotype *ap*-Gal4/+;Bnl-LexA lexOp-mCherry:CAAX/UAS-CD4:GFP^{11}, lexOp-Bnl^{WT}GFP$^{11(7x)}$. Max projections of GFP fluorescence of reconstituted Bnl:GFP (A,E,I,M) and of mCherry-marked membranes (C,G,K,O) in Bnl-expressing cells. Confocal sections with GFP fluorescence of reconstituted Bnl:GFP (C,G,K,O) and of mCherry-marked membranes (D,H,L,P) in Bnl-expressing cells. Scale bar, 40 μm.
